# Properties, Physiological Functions and Involvement of Basidiomycetous Alcohol Oxidase in Wood Degradation

**DOI:** 10.3390/ijms232213808

**Published:** 2022-11-09

**Authors:** Anna Pawlik, Sylwia Stefanek, Grzegorz Janusz

**Affiliations:** Department of Biochemistry and Biotechnology, Institute of Biological Sciences, Maria Curie-Skłodowska University, Akademicka 19 St., 20-033 Lublin, Poland

**Keywords:** alcohol oxidase, white rot, brown rot, fungi, lignin

## Abstract

Extensive research efforts have been devoted to describing yeast alcohol oxidase (AO) and its promoter region, which is vastly applied in studies of heterologous gene expression. However, little is known about basidiomycetous AO and its physiological role in wood degradation. This review describes several alcohol oxidases from both white and brown rot fungi, highlighting their physicochemical and kinetic properties. Moreover, the review presents a detailed analysis of available AO-encoding gene promoter regions in basidiomycetous fungi with a discussion of the manipulations of culture conditions in relation to the modification of alcohol oxidase gene expression and changes in enzyme production. The analysis of reactions catalyzed by lignin-modifying enzymes (LME) and certain lignin auxiliary enzymes (LDA) elucidated the possible involvement of alcohol oxidase in the degradation of derivatives of this polymer. Combined data on lignin degradation pathways suggest that basidiomycetous AO is important in secondary reactions during lignin decomposition by wood degrading fungi. With numerous alcoholic substrates, the enzyme is probably engaged in a variety of catalytic reactions leading to the detoxification of compounds produced in lignin degradation processes and their utilization as a carbon source by fungal mycelium.

## 1. Introduction

Thirty years ago, based on sequence similarities of four enzymes from evolutionary distinct organisms, Cavener [1] defined a new group of homologous proteins and called them glucose-methanol-choline (GMC) oxidoreductases. The author analyzed *Drosophila melanogaster* glucose dehydrogenase, *Escherichia coli* choline dehydrogenase, *Aspergillus niger* glucose oxidase, and *Hansenula polymorpha* methanol oxidase. Interestingly, these enzymes not only belong to different taxonomic groups but also catalyze different chemical reactions. Nevertheless, Cavener managed to identify a common pattern in their structure: a canonical ADP-binding βαβ-fold close to their amino termini, which is found in other flavoenzymes as well. Since then, a number of enzymes have been assigned to the GMC oxidoreductase family, expanding not only the number of proteins identified in many new taxa but also the number of new substrates for these oxidoreductases. The family comprises both oxidases and dehydrogenases, which are able to oxidize various sugars, alcohols, cholesterol, or choline, employing oxygen or different quinones, phenol radicals, or metal ions as the final or alternative electron acceptors [2,3,4]. Currently, it is known that all GMC enzymes include: (1) the FAD-binding domain composed of four separate subregions containing ADP-binding βαβ (discovered in 1992 by Cavener), (2) GMC pattern-2 (PROSITE PS00624), (3) GMC oxidoreductase pattern-1 (PROSITE PS00623), and (4) the C-terminal region [5]. Investigations of fungal enzymes belonging to the GMC family revealed the presence of seven subfamilies grouped into five clusters: AO (alcohol oxidase), CDH (cellobiose dehydrogenase), PO (pyranose oxidase), GO-GDH (glyoxal oxidase—glucose dehydrogenase), and AAO-PDH (aryl-alcohol oxidase—pyranose dehydrogenase) [2,5]. Further studies of the AO cluster resulted in the separation of the enzymes into five clades, three of which (basidiomycetous, ascomycetous, and saccharomycetous) are already being analyzed, whereas the other two (named AO-like from Basidiomycota and Ascomycota) are hardly known to science. Besides the medium sequence similarity, AO-like proteins are likely to oligomerize in a different pattern than typical alcohol oxidases [2]. Studies on the C-terminus suggest that AO from *Saccharomycetes* is localized in peroxisomes. A similar sequence (PTS—peroxisomal targeting signal) is also found in some other Ascomycota. In contrast, alcohol oxidase in Basidiomycota tends to associate with the hyphal periplasmic space and cell walls or is even secreted extracellularly [2,6]. In the case of *Pyricularia oryzae* alcohol oxidase, the presence of the cell wall integrity and stress response component (WSC) domain, which helps this enzyme adhere to xylans/chitin/glucan, has been proved [7]. In *Gleophyllum trabeum*, alcohol oxidase localized extracellularly is associated with the periplasmic external membranous system, probably due to the differences in the signal peptide sequences, compared to those found in yeasts [6]. The different cellular localization in *Basidomycota* may imply an alternative role of alcohol oxidase in this taxonomic group. All these fungal GMC enzymes are important for these organisms, considering their ability to oxidize simple compounds as carbon sources. Some of these substrates (methanol, glucose) may even be assimilated as a single carbon source in the medium during the cultivation of fungi in laboratory conditions. A limited number of yeast species (belonging to four genera *Hansenula (Pichia)*, *Ogataea*, *Candida*, and *Torulopsis*) are able to grow only on methanol as a sole carbon and energy source [8,9]. Upon encountering methanol in their ecological niches, they have developed sophisticated metabolic pathways not only to neutralize this toxin but also to catabolize this simple alcohol [10]. The initial methanol utilization reaction takes place in peroxisomes, which, as mentioned above, include alcohol oxidase, i.e., a key enzyme in this process. These organelles are strongly induced when cells are exposed to methanol; hence, these yeasts are frequently used as model organisms to study peroxisome biogenesis and function. In the first step of utilization, methanol is oxidized to formaldehyde and hydrogen peroxide by alcohol oxidases, and the resulting H_2_O_2_ is broken down to oxygen and water by catalase [9]. It should be underlined that fungal alcohol oxidase is able to oxidize not only methanol but also other simple aliphatic or aromatic compounds and other alcohols such as glycerol [6,11,12]. This ability broadens the range of substrates that should be removed/detoxified from the mycelium environment or assimilated as carbon sources. A similar function has been assigned to laccase, whose primary role is to protect the cell from hazardous compounds [13]. Initially, alcohol oxidase in the wood decay fungus *Phanerodontia chrysosporium* was described as part of a group of enzymes that produce hydrogen peroxide for peroxidases capable of lignin degradation [11]. Recent advances in the field of enzymology, molecular biology, anatomy, and ecology have provided a better understanding of the sophisticated methods of wood degradation used by different fungal organisms [14,15,16,17]. Despite the growing number of papers highlighting the role of alcohol oxidase in wood degradation, their findings have inspired a deeper analysis of its biological function once again.

## 2. Fungal Alcohol Oxidase—Occurrence in Nature, Structure, and Kinetics

As mentioned above, most of the alcohol oxidases (AO; EC 1.1.3.13) characterized so far originate from methylotrophic yeasts (e.g., *Hansenula, Pichia*, and *Candida*) and are located in their peroxisomes [18]. Moreover, this enzyme was also described in a limited number of wood rotting fungi, e.g., *Poria contigua*, *Phlebiopsis gigantea*, *G. trabeum*, and *P. chrysosporium*, or in other than yeast ascomycetous species such as *Penicillium* sp. or *Aspergillus* sp. [6,11,19,20,21,22]. It seems that most of the characterized basidiomycetous AOs are homooctamers composed of protein subunits with a molecular mass ranging from 72.4 to 79 kDa, depending on the species (Table 1). However, in *P. chrysosporium*, a homodimeric form of AO composed of 75 kDa subunits was described [11]. In certain ascomycetous fungi, a tetramer or a hexamer can be found [23,24]. The smallest monomer of alcohol oxidase described so far (13 kDa) was found in *A. terreus* as part of a heteropentamer including other proteins with MW equal to 85, 63, 43, and 27 kDa [25]. Only three wood-rotting fungal species (*P. chrysosporium*, *G. trabeum*, and *P. gigantea*) have been analyzed to determine the enzyme pI value, whose range appeared to be very narrow, i.e., 5.3–5.4 [6,11,20]. Most of the characterized AOs have a pI ranging from 4.3 to 6.1, except for *A. terreus*, which is 8.3–8.5 [25,26,27]. As a member of the GMC family, alcohol oxidase uses the non-covalently bound cofactor FAD. Each *P. chrysosporium* AO monomer is composed of two domains typical for GMC oxidoreductases: a substrate-binding domain and a FAD-binding domain. The former consists of a six-stranded β-antiparallel sheet, while the latter includes the typical Rossmann fold, featuring a sandwich of five-stranded parallel and three-stranded antiparallel β-sheets [28]. The same structure was proposed for *P. pastoris* alcohol oxidase by Vonck, et al. [29]. Moreover, AO in this methylotrophic yeast may include a modified FAD (the absolute configuration of carbon 2′ of the sugar chain attached to the isoalloxazine ring is changed from R to S in its active center). This substantial change results in slight decreases in the V_max_ value but significantly reduces the K_m_ value of the enzyme for the methanol substrate [30,31]. AO from *P. chrysosporium* was proved to oxidize simple aliphatic alcohols (from methanol to pentanol) and glycerol (17% of initial activity against methanol) [11]. Recombinant alcohol oxidase from *P. chrysosporium* expressed in *E. coli* was capable of enantioselective oxidation of diols [32]. This enzyme was also engineered to enhance its power towards glycerol [28] and was used to oxidize a range of diols and aromatic alcohols [33]. Similarly, metabolic capabilities were proved for AO from *P. contigua*. Besides aliphatic alcohols, the enzyme was able to oxidize formaldehyde or 2-mercaptoetanol [19]. Alcohol oxidase isolated from *G. trabeum* not only was able to catalyze the oxidation of simple aliphatic alcohols, diols, or benzyl alcohol, but also exhibited high activity against allyl alcohol (93% of the relative activity against methanol). The enzyme slightly oxidized D-glucose or D-arabinitol [6]. Similarly, AO from *P. gigantea* is hardly able to oxidize ribitol or erythritol [20]. This indicates the complementary activities of AO to those of aryl-alcohol oxidase, which prefers benzyl alcohol and a range of aromatic alcohols as substrates and is concurrently unable to catalyze the oxidation of simple aliphatic alcohols [34]. Moreover, it should be noticed that the broad substrate specificity is typical for LME (lignin-modifying enzymes) [35], as they are engaged in the decomposition of the lignin heteropolymer. The pH optimum of alcohol oxidase tends to be rather neutral or even alkaline [6,28], in contrast to ligninolytic enzymes having pH optima in acidic values: laccase-4.5–5 [36] or manganese peroxidase-4.5 [37]. Interestingly, a similarly neutral pH optimum characterizes aryl-alcohol oxidase [38,39], which among others is supposed to cooperate in the extracellular environment. Alcohol oxidase activity in wood-degrading fungi was proved to be inhibited by ions (Cu^2+^, Fe^2+^) [20], whereas AOs from yeast and other ascomycetous fungi are also affected by various compounds: alcohols [40], DMSO [41], or formaldehyde [42], H_2_O_2_ [40]. However, it seems that the lack of determined inhibitors of alcohol oxidases from wood-rotting fungi is associated with the insufficient number of detailed studies thereof rather than the properties of the enzymes.

## 3. Gene Structure and Regulation of Expression

Although AO was identified almost 60 years ago, only a limited number of genes encoding this enzyme in fungi have been cloned and characterized so far [6,11,28]. With the recent interest in lignocellulose degradation, an increasing number of genes and transcripts coding for putative alcohol oxidase have been revealed in many fungal genomes and transcriptomes by numerous sequencing projects led by multi-institutional consortia (e.g., 1000 Fungal Genomes Project https://mycocosm.jgi.doe.gov/mycocosm/home/1000-fungal-genomes) accessed on 1 May 2022 [5,46,47,48,49], which suggests that genes coding for AO are widely distributed in nature. Corresponding genes were also found in the genomes, transcriptomes, and proteomes of many white and brown rot fungi, i.e., *Agaricus bisporus* [47], *Rigidoporus microporus* [49], *P. gigantea* [50], *G. trabeum* [51], *Coprinopsis cinerea* [52], *Rhodonia (Postia) placenta* [46], *Trametes versicolor* [53], *Cerrena unicolor* [54], *P. carnosa*, and *P. chrysosporium* [48]. As a result, up to six alcohol (methanol) oxidase-encoding genes (AA3_3 CAZyme family) were identified in the genomes of the brown rot *R. placenta* [46] and up to 14 genes were found in the white rot *Steccherinum ochraceum* [55]. The first heterologous expression of an alcohol oxidase originating from the wood decay fungus *G. trabeum* was reported in 2007, which also makes it one of the best-characterized *mox* genes in this ecological group of fungi. Its DNA sequences showed an open reading frame of 1953 bp coding for 651 amino acids [6]. As demonstrated, the *mox* gene of *G. trabeum* and other Basidiomycota does not contain a typical signal peptide [6]. Recently, a novel *aox1* gene was identified, isolated [11], and expressed in *E. coli* [28]. The corresponding gene does not include the C-terminal sequence involved in targeting yeast AOs to peroxisomes, as expected for basidiomycete AO [11]. As shown in Table 2, putative general transcription (one TATATA and up to six CAAT boxes) and response elements to metals (MRE), xenobiotics (XRE), heat-shock (HSE), stress (STRE), and signals for regulatory proteins implicated in oxidative stress (AP-1), regulation by nitrogen (AP-2, NIT), induction by copper (ACE-1), regulation of transcription (Sp1), and carbon sources (CreA) were detected in the promoter region upstream of the sequence of the *mox* gene in some representatives of wood-decaying fungi. Interestingly, similar regions were identified in the promoter regions of other fungal genes coding for lignocellulose-degrading enzymes [56]. Furthermore, regulatory elements of the response to carbon, nitrogen, heat-shock, and xenobiotics were identified in all analyzed fungal transcriptomes (Table 2). In turn, the promoter that controls alcohol oxidase expression in methylotrophic yeasts, known to be one of the strongest promoters in nature, is at the same time one of most tightly controlled yeast promoters [18]. Three transcription factor genes TRM1, TRM2 (involved in methanol-specific activation and derepression, respectively), MPP1 (encodes a Zn(II)2Cys6-type transcription factor), and Mig1p (involved in glucose repression) were proved to be involved in AO promoter regulation in methylotrophic yeasts [57].

AO is an abundant protein, and its synthesis is strictly regulated by repression/derepression and induction mechanisms that occur at the transcriptional level. Various aspects of its sorting and assembly/activation render AO a unique protein [18]. It has been demonstrated that catabolite repression of *aox* by glucose is generally observed in a wide range of methylotrophic yeast species. However, the catabolite repression of the alcohol oxidase gene by glycerol is species-specific, and the profile of catabolite repression does not seem to reflect taxonomy but may reflect the environment inhabited by each yeast species [57,58,59].

For decades, fungal genes encoding ligninolytic enzymes involved in natural wood decay have been found to be differentially regulated in response to a wide variety of environmental signals. In white rot fungi, the expression of lignolytic enzymes is generally triggered by nutrient depletion during secondary metabolism, although differential responses to C/N ratios and even to the presence of a lignocellulosic substrate have been observed among individual enzymes and fungal species. Furthermore, *cis*-acting elements related to metal and xenobiotic response mechanisms and temperature shock or oxidative stress responses have been identified in the promoter regions of those genes, as mentioned above [56]. The production of extracellular laccase and aryl-alcohol oxidase by *P. eryngii* in a liquid medium containing ammonium tartrate (non-limiting nitrogen conditions) has also been reported [60]. An intracellular alcohol oxidase with distinct glycerol oxidase activity was isolated from the white rot basidiomycete *P. chrysosporium* grown on L-lactate induction medium. Unexpectedly, neither glycerol nor glucose or such alcohols as methanol, ethanol, or 1-propanol, acted as inducers of AO in this strain [11], in contrast to strongly methanol-inducible genes, such as AOX1, in the methylotrophic yeast *O. minuta*. A recent study revealed that the *O. minuta* AOX1 promoter (P_AOX1_) is induced on methanol and repressed on glucose and glycerol. P_AOX1_ remained repressed when methanol was present in addition to glucose or glycerol, indicating that glucose and glycerol cause a strong catabolite repression effect [57,61]. It was also proved that three transcription factor genes TRM1, TRM2, and MPP1 are involved in AOX1 promoter regulation in *O. minuta* in two different pathways, which compensate for each other [57]. In turn, proteomic studies in *T. versicolor* grown in tomato juice supplemented with CuSO_4_ and MnCl_2_ and in *T. trogii* grown in minimal media detected peptides corresponding to GMC oxidoreductases, including one aryl-alcohol oxidase. Additionally, two methanol oxidases were found in *T. trogii* [62,63]. The analysis of the transcriptome of *Pycnoporus sanguineus* BAFC 2126 grown at the stationary phase in media supplemented with CuSO_4_ showed the presence of transcripts (Psang07044 and Psang01120) encoding putative alcohol oxidases. Translated ORFs showed homologies (68% and 82%, respectively) with an aryl-alcohol oxidase-like protein from *T. versicolor*. However, seven of them showed a high homology with sequences encoding putative alcohol (methanol) oxidases of *T. versicolor* and *D. squalens* [64].

## 4. Involvement of Alcohol Oxidase in Wood Degradation

**Figure 1:**
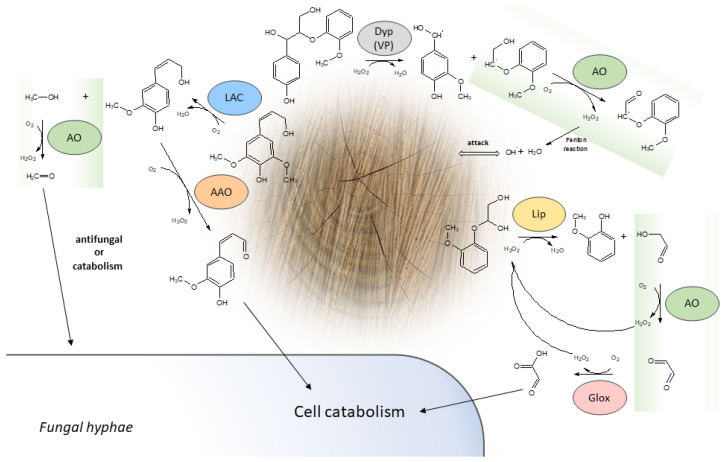
Comparison of lignin degradation by peroxidases and laccase with the involvement of alcohol oxidase, aryl-alcohol oxidase, and glyoxal oxidase [5,73,80,81,82]. AO—alcohol oxidase, AAO—aryl-alcohol oxidase, MnP—manganese peroxidase, VP—versatile peroxidase, LiP—lignin peroxidase, DyP—dye-decolorizing peroxidase, GLOX—oxalate oxidase. Other enzymes capable of catalyzing similar reactions are presented in parentheses.

It has been evidenced that, due to the synthesis of hydrogen peroxide, i.e., a crucial substrate for heme peroxidases in lignin degradation, the role of fungal alcohol oxidase in wood decomposition may be classified as that of lignin-degrading auxiliary enzymes (LDA). Among these enzymes, there are aryl-alcohol oxidase, glyoxal oxidase, cellobiose dehydrogenase, and pyranose 2-oxidase. Hydrogen peroxide is also a key factor in the Fenton reaction, which is mainly used in the wood degradation strategy by brown rot fungi [35]. LDA enzymes secreted extracellularly by fungi are able to oxidize a range of substrates involved in wood degradation and thus serve as a carbon source in the fungal metabolism. Considering the ability of alcohol oxidase to oxidize different aliphatic or aromatic alcohols [6,11,12], its extracellular form may be involved in secondary lignin degradation by oxidizing reaction products formed by laccase or heme peroxidases (Figure 1). Laccase is known to cause lignin demethoxylation [66]. The resulting methanol is further oxidized by AO to formaldehyde, which is then probably transported into the cell and metabolized [67]. It has already been proved that methanol is produced in cultures of both white and brown rot fungi [6,68,69]. Moreover, certain bacteria and fungi can catalyze the demethylation of lignin by *O*-demethylases; thus, methanol is released by different organisms in natural consortia causing wood degradation [70]. Most probably, this is why alcohol oxidase is found in most wood-degrading fungi even if they lack laccases or heme peroxidases [50]. Additionally, it seems that formaldehyde formed in the AO-catalyzed reaction may serve as a protective agent against unwanted and harmful organisms in the wood degrading community [71]. However, it should be noticed that certain ascomycetous fungi are also capable of degrading formaldehyde [72]. As described by Chen, et al. [73], lignin peroxidase (LiP) can catalyze the homolytic C_α_-C_β_ cleavage in a series of reactions finally producing hydroxy aldehyde, which may be further oxidized by alcohol oxidase to glyoxal—a substrate for glyoxal oxidase (GLOX) also producing hydrogen peroxide as a by-product (Figure 1). Similar cleavage reactions may be catalyzed by dye decolorizing peroxidase (DyP) or versatile peroxidase (VP), and the resulting aromatic alcohols are potential substrates for AO [35]. Moreover, oxalate synthesis proceeding in the proposed way may be important in the regulation of the concentration of manganese ions (crucial for manganese peroxidase activity) and iron ions (required for Fenton reaction) by lowering the pH of the close extracellular environment of the mycelium (laccase and peroxidases have acidic pH optima) [74]. It should be mentioned that the AO-induced hydrogen peroxide synthesis may be insufficient, compared to the iron-dependent hydroquinone autooxidation in brown rot fungi [75]. An analysis of AO promoter regions derived from different wood degrading fungi showed similar regulative elements to these found in genes coding for laccase or POD (Table 2) [56]. Furthermore, an analysis of the *C. unicolor* transcriptome proved that the expression of the two AO genes was up-regulated when the fungus was grown on ash, birch, or maple sawdust. However, it should be noticed that two other genes were down-regulated in the same culture conditions, implicating different functions of these enzyme isoforms [54]. This may be supported by the fact that one of the AO genes was over expressed when the fungus was cultivated in white light [76]. In a similar analysis, Henske, et al. [77] proved a 35-fold increase in the AO gene expression and a 285-fold increase in the peroxidase gene expression in *Pycnoporus cinnabarinus* when the fungus was grown on plant material. The authors suggested that the production of these enzymes was triggered by lignin itself or by the products of its breakdown. Hori et al. [50] showed that several genes of alcohol oxidase (in the paper named methanol oxidase) are present in different fungal species, whereas some LME are absent, probably depending on the fungal lignin degradation strategy. They also proved that the expression of three genes coding for alcohol oxidase (especially one of them) in *P. gigantea* was up-regulated when the fungus was cultivated on lodgepole pine. Similarly high expression of an enzyme with over 85% similarity to AO from *G. trabeum* was observed during fungus cultivation on medium containing only cellulose, which may implicate that the regulation of the expression of this alcohol oxidase is related to polymer degradation [46]. Among enzymes generating hydrogen peroxide, alcohol oxidase in *G. trabeum* was highly up-regulated when the fungus was grown on both cellulose and cedar wood, suggesting its crucial role in brown rot wood degradation [51]. Alcohol oxidase corresponding genes were also overexpressed in *P. chrysosporium* and *P. placenta* (Pchr126879 and Ppl118723, respectively) secretomes in cultures on ball-milled aspen and glucose media. The identified sequences appeared to be highly conserved (>87% identical to the methanol oxidase from the brown rot fungus *G. trabeum*) [6,78]. In turn, the methanol oxidase transcript levels in another brown rot fungus *Wolfiporia cocos* ortholog (Wolco_24953) were unaffected when the fungus was grown on woody substrates [79].

## 5. Conclusions

Scientific data have suggested that basidiomycetous alcohol oxidase is important in secondary reactions during lignin decomposition by wood degrading fungi. With numerous alcoholic substrates, the enzyme is probably engaged in a variety of catalytic reactions leading to the detoxification of compounds produced in lignin degradation processes and their utilization as a carbon source by fungal mycelium. Hydrogen peroxide, i.e., the by-product of alcohol oxidation by AO, serves as a substrate for heme peroxidases (LiP, MnP, VP, and DyP) or is engaged in Fenton reactions, all being crucial parts of the sophisticated wood degrading machinery. Moreover, it seems that formaldehyde produced by alcohol oxidase may be used by fungi as biocontrol agents against unwanted organisms in their ecological niche.

Still, only few basidiomycetous AOs have been purified and characterized in terms of their physicochemical and kinetic properties. Little is known about their cellular localization (except for *G. trabeum*) in both white and brown rot fungi. As demonstrated, alcohol oxidase genes have been shown to be overexpressed in response to lignin substrates in the medium; however, it is not clear whether their expression is boosted via regulatory elements in their promoter regions or whether their genes are clustered with other wood-degrading enzymes and are thus expressed together. Future studies consisting of detailed enzyme analyses, mapping of lignin degradation pathways, and the regulation of gene expression may highlight the complete physiological role of the enzyme in wood-degrading fungi.

## Figures and Tables

**Table 1:** Known alcohol oxidases from wood-rotting fungi.

Fungus Name (Type of Wood Rot)	pH	pI	MW (kDa)	K_m_ (mM) vs. Methanol	Other Substrates	References
*Gloeophyllum trabeum* (BR ^1^)	6.0–10.0	5.3	628.0 (8 × 72.4)	2.3	ethanol allyl alcohol 1-propanol 1-butanol 1-pentanol 2-methyl-1-propanol 3-methyl-1-butanol 2-propanol 2-butanol 3-pentanol 2-butene-1,4-diol benzyl alcohol 4-hydroxybenzyl alcohol D-arabinitol D-glucose ethanolamine	[6]
*Phanerodontia chrysosporium* (WR ^2^)	6.0–10.5	5.4	2 × 75.0 or 4 × 75.0	0.785–36.6	ethanol (2-hydroxyethoxy)ethanol 1-propanol isopropanol 1,2-propanediol 5-aminopentan-1-ol butane-1,4-diol 1-pentanol pentane-1,4-diol pentane-1,5-diol hexane-1,6-diol hexane-1-ol octane-1,8-diol glycerol diethylene glycol 2,2’-sulfanediyldi(ethan-1-ol) 2,2’-[ethane-1,2-diylbis(oxy)]di(ethan-1-ol) ethylene glycol ethylene glycol mono-methylether	[11,43,44]
*Phlebiopsis gigantea* (WR ^2^)	7.3–9.0	5.3	576.0 (8 × 72.5)	1.8	allyl alcohol D-ribose erythritol ethanol isopropanol 1-butanol 1-propanol ribitol	[20]
*Polyporus obtusus, Radulodon casearius* (WR ^2^)	6.5–9		300.0			[45]
*Poria contigua* (BR ^1^)			610.0 (8 × 79.0)	0.2	ethanol 1-propanol 1-butanol isopropyl alcohol 2-propin-1-o1 formaldehyde 2-mercaptoethanol	[19]

^1^ BR—brown rot fungus. ^2^ WR—white rot fungus.

**Table 2:** Location of regulatory elements in alcohol oxidase promoter sequences (https://mycocosm.jgi.doe.gov/ accessed on 1 May 2022).

Fungus	TATA	CAAT	ACE-1	MRE	HSE	XRE	Cre-A	STRE	NIT2	AP1/ AP2	Sp1
*Agaricus bisporus* var *bisporus* (H97) protein ID 195553 [47]	−80	−96 −230 −1279 −1312 −1357	−240 −2418 −2920 −3066		−23	−2881	−938 −2503 −2625	−311 −720 −1104	−135 −267 −526 −763 −2772 −2805		
*Cerrena unicolor* protein ID 352889	−49	−235 −353 −399 −418 −425	−710 −742 −753 −1066 −1484	−627	−53	−1287 −1309	−549 −662 −669 −1526 −1825 −1923		−172 −344 −414 −639 −1289 −1632	−499 −862	−799
*Phanerochaete chrysosporium* RP-78 protein ID 6010	−75	−489 −514 −632 −1743 −1899	−673	−1985 −2007	−26	−1841 −1929	−826 −987 −1110 −1330 −1382 −1452		−936 −1814 −2454	−1642	−828 −1015 −1027 −2132
*Gloeolophyllum trabeum* protein ID 139980 [53]	−96	−508 −579 −857 −1281 −1410 −1763	−119 −565 −971 −1331 −2659		−25	−1691 −2556	−871 −965 −2341 −2369		−1258 −1386 −1487		−273 −412 −636 −1432 −1640 −1668 −1987 −2270 −2306
*Postia placenta* MAD-698-R-SB12 protein ID 1045608 [65]	−43	−393 −565 −786 −946 −966 −1321	−218 −717		−16	−416 −886 −1227 −1601	−263 −445 −672 −1174 −1612 −1746 −1883 −1901 −2149 −2301			−2148	−364 −1011 −1614 −2056 −2423
